# Genetic associations with socioeconomic status are limited in explaining the socioeconomic health gradient

**DOI:** 10.1073/pnas.2505061122

**Published:** 2025-05-16

**Authors:** David Füller, Benjamin Sasko, Oliver Ritter

**Affiliations:** ^a^Department of Internal Medicine, Division of Cardiology, University Hospital Brandenburg an der Havel 14770, Germany; ^b^Brandenburg Medical School (Theodor Fontane), Brandenburg 14770, Germany; ^c^Faculty of Health Sciences Brandenburg, Brandenburg 14770, Germany; ^d^Medical Department II, Division of Cardiology, Marien Hospital Herne, University Hospital of the Ruhr-University Bochum, Herne 44625, Germany

Wu et al. (1) identified genetic variants associated with indicators of socioeconomic status (SES), i.e., level of education, household income, occupational status, and regional socioeconomic deprivation, using whole-exome sequencing in 350,000 individuals enrolled in the United Kingdom Biobank. The group also found associations of the identified variants and several phenotypic traits and health conditions. Here, we want to draw attention toward three aspects.

First, the authors did not find an association between SES-associated variants and coronary heart disease (CHD) or cancer, while it is known that socioeconomic disparities play a significant role in these diseases (2–4). Although the study shows associations of SES-associated variants with some cardiovascular risk factors (diabetes, social isolation, obesity, and chronic renal failure), it does not show associations with others, in particular arterial hypertension and hyperlipoproteinemia that both have a strong genetic underpinning. Other studies have challenged the assumption that health disparities in CHD and cancer can be well explained by differences in the prevalences of risk factors (5–7). Given the absence of direct associations between SES-associated variants and CHD or cancer and considering that the observed associations with certain risk factors are unlikely to fully account for the socioeconomic gradient, we conclude that the lack of association with CHD and cancer substantially weakens the concept that genetic mechanisms play an important role in driving health disparities at the population level.

Second, in our opinion, the article has fallen too short in placing the presented evidence and its rationale in context of the evidence that has only been referred to as “genetic and environmental pathways”. To the other readers of PNAS, we would like to point at the existing evidence on the biological correlates of lower SES: These biological correlates have been shown to include immunomodulation, clinically relevant inflammation, changes in transcription, and DNA methylation signatures (8–10). Previous investigations of the “biology of (socioeconomic) adversity” have been understood as studies of the biological consequences of social and economic inequality and considered how individuals access resources to cope with stress. Neuroticism, which the authors found to be associated with SES-related genetic variants, suggests that genetic factors may indeed represent a biological mechanism along these same lines, as it might correlate with an increase in inflammation through an increase in perceived stress.

Third, the authors conclude that there are gene–environment interactions that are relevant for the observed association of SES and genetic variants. We believe that there are two possible explanations for the increasing *P*-values observed when adjusting for geography. First, certain SES-associated variants may be more prevalent in specific regions, in the sense of genetic clustering, and could have a greater significance for SES in those areas. This can lead to increasing *P*-values after adjustment. Second, using Middle-Layer Super Output Areas for adjustment for geographical regions which can be very heterogenic in terms of their inhabitants and socioeconomic setting may introduce model overfitting. This could account for the increase in *P*-values when adjusting for geography in the model on regional socioeconomic deprivation.
